# Prinsepiae Nux Extract Activates NRF2 Activity and Protects UVB-Induced Damage in Keratinocyte

**DOI:** 10.3390/antiox11091755

**Published:** 2022-09-05

**Authors:** Shih-Han Wang, Yi-Siao Chen, Kuei-Hung Lai, Chung-Kuang Lu, Hsun-Shuo Chang, Ho-Cheng Wu, Feng-Lin Yen, Lo-Yun Chen, Jin-Ching Lee, Chia-Hung Yen

**Affiliations:** 1 Graduate Institute of Natural Products, College of Pharmacy, Kaohsiung Medical University, Kaohsiung 80708, Taiwan; 2 National Natural Product Libraries and High-Throughput Screening Core Facility, Kaohsiung Medical University, Kaohsiung 80708, Taiwan; 3 Ph.D. Program in Environmental and Occupational Medicine, College of Medicine, Kaohsiung Medical University and National Health Research Institutes, Kaohsiung 80708, Taiwan; 4 Ph.D. Program in Clinical Drug, Development of Herbal Medicine, College of Pharmacy, Taipei Medical University, Taipei 11031, Taiwan; 5 Graduate Institute of Pharmacognosy, College of Pharmacy, Taipei Medical University, Taipei 11031, Taiwan; 6 National Research Institute of Chinese Medicine, Ministry of Health and Welfare, Taipei 11221, Taiwan; 7 School of Pharmacy, College of Pharmacy, Kaohsiung Medical University, Kaohsiung 80708, Taiwan; 8 Drug Development and Value Creation Research Center, Kaohsiung Medical University, Kaohsiung 80708, Taiwan; 9 Department of Fragrance and Cosmetic Science, College of Pharmacy, Kaohsiung Medical University, Kaohsiung 80708, Taiwan; 10 Department of Marine Biotechnology and Resources, National Sun Yat-sen University, Kaohsiung 80424, Taiwan

**Keywords:** NRF2, natural product, Shen Nong Ben Cao Jing, ROS, UVB irradiation, antioxidant

## Abstract

Ultraviolet B (UVB) is one of the most important environmental factors that cause extrinsic aging through increasing intracellular reactive oxygen species (ROS) production in the skin. Due to its protective roles against oxidative stress, nuclear factor erythroid-2-related factor (NRF2) has been traditionally considered as a target for skin aging prevention. Here, we identified the extract of Prinsepiae Nux, a top-grade drug listed in Shen Nong Ben Cao Jing, as a potent NRF2 activator by high-throughput screening. A bioassay-guided fractionation experiment revealed that NRF2-activating components were concentrated in the 90% methanol (MP) fraction. MP fraction significantly increased the expression of NRF2 and HO-1 protein and upregulated HO-1 and NQO1 mRNA expression in HaCaT cells. Moreover, MP fraction pre-treatment dramatically reversed UVB-induced depletion of NRF2 and HO-1, accumulation of intracellular ROS, NF-κB activation, and the upregulation of pro-inflammatory genes. Finally, the qualitative analysis using UPLC-tandem mass spectroscopy revealed the most abundant ion peak in MP fraction was identified as α-linolenic acid, which was further proved to activate NRF2 signaling. Altogether, the molecular evidence suggested that MP fraction has the potential to be an excellent source for the discovery of natural medicine to treat/prevent UVB-induced skin damage.

## 1. Introduction

Skin aging is a natural phenomenon that happens to every human body. It can be divided into extrinsic aging and intrinsic aging. Intrinsic aging is an inevitable chronological process influenced by internal physiological factors and characterized by the reduction in proliferative cells in the basal layer and the decrease in nutrition supply for dermis and epidermis [1]. Extrinsic aging is triggered by external environmental factors such as ultraviolet rays (UV), alcohol consumption, smoking, air pollution, malnutrition, or even mental stress [2]. The exposure of skin to UVB (280–320 nm) is one of the main causes that can accelerate skin aging, which is characterized by thinning, dyspigmentation, wrinkling, loss of elasticity, sagging, laxity, and dryness [2]. UVB has high photon energy and can penetrate the epidermis and cause a series of immediate effects, such as redness and inflammation [3]. Under long-term or high-dose exposure, UVB increased skin cancer risk by increasing DNA damage and mutations [4,5]. UVB also leads to excessive production of reactive oxygen species (ROS) that activate plenty of signaling pathways that accelerate skin aging [6]. UVB-induced excessive ROS production could lead to telomere shortening/mutations and subsequent cell death or senescence of cells in the basal layer. Chronic, low-grade inflammation is also a major contributor to the skin aging process. UVB overexposure causes oxidative stress in epidermal cells, which in turn results in damaged cells and triggers inflammatory response. In addition, ROS could induce the nuclear factor kappa light chain enhancer of activated B cells (NF-κB) to translocate to the nucleus, where it can upregulate pro-inflammatory genes, such as interleukin 6 (IL-6) and cyclooxygenase-2 (COX-2) and cause skin inflammation and redness [7]. Therefore, preventing the skin from UVB-induced oxidative stress is a crucial issue.

One of the most important cellular antioxidative stress responses is the nuclear factor erythroid-2-related factor (NRF2) signaling pathway. NRF2 is a transcription factor belonging to the Cap ‘n’ collar family. In the cytoplasm, NRF2 combines with Kelch-like ECH-associated protein 1 (Keap1) as an inactive complex, which allows NRF2 to be polyubiquitinated and subsequently degraded by the proteasome [8]. However, exposure to ROS and electrophilic stress can modify specific cysteine residues in Keap1, causing a conformational change in the Keap1-Cul3-E3 ubiquitin ligase, preventing NRF2 ubiquitination and degradation [8,9]. NRF2 will next translocate to the nucleus and bind to the Maf protein to form a complex protein. This complex will further bind to the antioxidant response elements (AREs) and finally drive the expression of antioxidant and detoxification genes, such as NAD(P)H:quinone oxidoreductase-1 (NQO1) and heme oxygenase-1 (HO-1). In addition to the antioxidative effect of NRF2, recent studies have also pointed out that NRF2 has an anti-inflammatory effect [10]. Accordingly, NRF2 activators are considered to protect skin from photoaging.

Shen Nong Ben Cao Jing is the earliest classic Traditional Chinese Medicine pharmacology. The top-grade drugs listed in Shen Nong Ben Cao Jing have been used for thousands of years and are considered non-toxic and have health benefits when consumed regularly. In this study, we identified a fraction extract of Prinsepiae Nux as a potent NRF2 activator by screening a fraction extract library prepared from 60 top-grade drugs. Prinsepiae Nux is the dried kernel of *Prinsepia uniflora* Batal. or *Prinsepia uniflora var. serrata* Rehd [11]. Prinsepia Nux has been used to treat acute conjunctivitis and acute keratitis in the practice of Traditional Chinese Medicine. The objective of this study was to further investigate the potential of Prinsepia Nux for the development of pharmaceutics and cosmetics for the treatment or prevention of photodamage.

## 2. Materials and Methods

### 2.1. Chemical Reagents

Dulbecco’s Modified Eagle’s Medium (DMEM), heat-inactivated fetal bovine serum (FBS), and H_2_DCF-DA were purchased from Thermo scientific (Madison, WI, USA). Resazurin was purchased from Cayman Chemical (Ann Arbor, MI, USA). EPIXTRACT Nuclear Protein Isolation Kit (ENZ-45016) was purchased from Enzo Life Sciences (Hines Dr, Ann Arbor, MI, USA). Hoechst H33258 was purchased from Sigma-Aldrich (Burlington, MA, USA). α-Linolenic acid (ALA) and γ-linolenic acid (GLA) were purchased from ChemScene LLC (Monmouth Junction, NJ, USA). All other chemicals and reagents used were of analytical grade.

### 2.2. Shen Nong Ben Cao Jing Top-Grade Drugs Library and the Extraction of Prinsepiae Nux

The Shen Nong Ben Cao Jing Top-Grade Drugs Library, a collection of 816 fractions prepared from 60 top-grade drugs, was established by Dr. Chung-Kuang Lu. Prinsepiae Nux was purchased from a local Traditional Chinese Medicine Store. Prinsepiae Nux (10 g) was ground into powder and extracted three times with 60% ethanol_(aq)_ (three days each) at room temperature to give the 60% ethanol extract (340 mg) on the removal of the solvent. The 60% ethanol extract was subjected to a standard solvent partition experiment. The sample was extracted with H_2_O and ethyl acetate (150 mL, 1:1 *v/v*) to obtain the H_2_O-partitioned (HP) fraction. The residual ethyl acetate was removed in vacuo using a rotary evaporator (160 mg remaining), then further extracted with 90% methanol_(aq)_ and hexane to obtain the 90% methanol (MP) fraction and hexane (HEP) fraction on the removal of the solvent. MP fraction was harvested (138 mg) and stored at −20 °C.

### 2.3. Cell Cultures

The immortalized human skin keratinocyte cell line (HaCaT cells) was cultured in DMEM with 10% FBS, penicillin (100 U/mL), and streptomycin (100 μg/mL). HEK293T cell line was cultured as described previously [12].

### 2.4. ARE-Luciferase Reporter Assay and Cell Viability Assay

HaCaT/ARE cell was established and cultured as described in our previous publication [13]. HaCaT/ARE cells were seeded (1 × 10^4^ cells/well) in a 96-well plate, then treated with indicated concentrations of the extract. After 14 h of treatment, 0.1 mg/mL of resazurin was added and further incubated for 4 h at 37 °C. Fluorescence of the reduced resazurin (ex/em: 530/590 nm) was measured from the culture supernatant by Synergy HT Multi-Mode Reader (BioTek, Winooski, VT, USA) to analyze cell viability. The cells were then harvested for luciferase activity measurements according to the manufacturers’ instructions (Promega Corporation, Madison, WI, USA) [14]. Relative luciferase activity was calculated by normalizing luciferase activity to cell viability. The average relative luciferase activity of DMSO wells was defined as the control and attributed to a relative NRF2 activity of 100%.

### 2.5. NRF2 Knock-Down Experiment

Two plasmids encoding different shRNAs for NRF2, namely shNRF2-1 (5′AGTTTGGGAGGAGCTATTATC, clone#: TRCN0000007555) and shNRF2-2 (5′GCTCCTACTGTGATGTGAAAT, clone#: TRCN0000273494); the control plasmids for the RNA interference (pLKO.1-shSCR); the packaging plasmid (pCMV-DR8.91); and the envelope plasmid (pMD.G), were obtained from National RNAi Core Facility (Academia Sinica, Taiwan). Pseudotyped lentiviruses preparation and infection to HaCaT/ARE cells were performed as described previously [12]. Lentivirus-infected cells were used to perform the ARE-Luciferase reporter assay as described in Section 2.4.

### 2.6. NF-κB-Luciferase Reporter Assay

The NF-κB reporter plasmid–pHAGE NFkB-TA-LUC-UBC-GFP-W was a gift from Darrell Kotton (Addgene plasmid #49343) and used for lentivirus preparation as described previously [12]. To generate stable cell lines, HaCaT cells were infected with the pseudotyped lentivirus-containing medium with the presence of polybrene (8 μg/mL) for 24 h. Cells were expanded, and then GFP-positive cells were sorted with MoFlo^®^ High-Performance Cell Sorter (Beckman Coulter, CA, USA) and named HaCaT/KBR cells. HaCaT/KBR cells were used to perform luciferase reporter assay as described in Section 2.4.

### 2.7. Immunoblot

Immunoblots were performed based on methods described in a previous paper [13]. The following antibodies were used: anti-NRF2 (GTX103322, GeneTex, Irvine, CA, USA), anti-GAPDH (60004-1g, Proteintech, Rosemont, IL, USA), anti-IκB alpha (12045-R116, Sino Biological, BDA, Beijing, China), anti-phospho- IκB alpha (#9246, Cell Signaling Technology, MA, USA), anti-alpha Tubulin (11224-1-AP, Proteintech, Rosemont, IL, USA), and anti-Lamin B1 (66095-1-Ig, Proteintech, Rosemont, IL, USA). Nuclear and cytoplasmic proteins were collected using EPIXTRACT Nuclear Protein Isolation Kit according to the manufacturer’s instructions.

### 2.8. Quantitative Real-Time PCR (QPCR)

QPCR was performed based on methods described in a previous paper [12]. The mRNA level was normalized with the GAPDH mRNA level. The primers used in this study are shown in Supplementary Table S1.

### 2.9. UVB Irradiation

Before the exposure to UV, the cells were pretreated with indicated extract or control solvent. After treatment, the cells were washed with PBS to remove residual DMEM and replenish a thin layer of PBS. Then, the HaCaT cells were exposed to UVB radiation at 30 mJ/cm^2^ using UVP^®^ CL-1000^®^ Ultraviolet Crosslinkers (UVP, Upland, CA, USA) equipped with five 8-watt UV-B G8T5E tubes at a distance of 3.5 inches from the light source.

### 2.10. Intracellular ROS Detection

The production of intracellular ROS was determined by H_2_DCF-DA. HaCaT cells were placed in the incubator to allow treatments to act for a specific time, followed by the replacement of 5 µM H_2_DCF-DA and incubated for 30 min. After UVB irradiation, DNA was stained with Hoechst H33258 to localize cell nuclei. Images for nuclei and ROS were acquired and analyzed automatically by an HCS instrument (ImageXpress Micro System, Molecular Devices, Sunnyvale, CA, USA).

### 2.11. Tandem Mass Spectrometry (MS/MS) Non-Targeted Fragment Ions Collection Using Ultra-Performance Liquid Chromatography Quadrupole Time-of-Flight Mass Spectrometry (UPLC-QTOF-MS)

The MS^2^ data collection was carried out based on a Waters SYNAPT G2 LC/Q-TOF (Waters Corporation, Milford, MA, USA) system. The chromatographic separation prior to the MS spectra was performed using a C18 column of Waters Acquity UPLC BEH (Waters, 1.7 µm, 2.1 mm × 100 mm). The mobile phase was prepared withn MeCN (A, containing 0.1% formic acid)/water (W, containing 0.1% formic acid) gradient sequences as follows: 0.01 min-60% A, 30 min-100% A (for HEP fraction); 0.01 min—3% A, 30 min—30% A (for HP fraction); and 0.01 min—10% A, 30 min—100% A (for MP fraction). The flow rate was set up at 0.5 mL/min, and the temperature of the column part was maintained at 40 °C in the oven. Additionally, 5 mg extracts were dissolved in 1 mL of methanol (4000 ppm) and filtered through a 0.45 μm membrane filter. The sample injection was executed automatically with a 4 μL volume per injection. The non-targeted MS^1^ and MS^2^ data were collected within the range of *m/z* 100–2000. The automated data-dependent acquisition (DDA) approach was applied in the MS^2^ scans, and non-targeted selections of 5 precursor ions were fragmented with ramping of the collision energy from 10–50 V. The acquired MS data were finalized by Waters MassFragment software (MassLynx4.1, Waters, Waters, Milford, MA, USA).

### 2.12. Global Natural Product Social (GNPS)-Based Molecular Networking(MN) Analysis

A GNPS web-based platform (https://gnps.ucsd.edu) was applied to analyze and output the MS/MS molecular networking data on 27 December 2021. The MS/MS spectra were window-filtered according to the top 5 strongest ion peaks in the ± 50 Da window throughout the spectrum. A network was then created, in which linkages between nodes were filtered by a cosine value above 0.70 and at least four matched peaks. Then, the appeared nodes in the network were annotated based on the experimental MS^2^ fragmentations of isolates. The molecular network was visualized and laid out using Cytoscape 3.8.2 (Cytoscape 3.8.2, San Francisco, CA, USA).

### 2.13. Statistical Analysis

GraphPad Prism 6.01 software (La Jolla, CA, USA) was used for data analyses. The results are presented as the mean ± standard deviation (mean ± SD). All studies’ data were analyzed using an analysis of variance, followed by Dunnett’s test for pair-wise comparisons. The statistical significance was defined as * *p* < 0.05, ** *p* < 0.01, and *** *p* < 0.001 compared with solvent control cells; # *p* < 0.05, ## *p* < 0.01, and ### *p* < 0.001 compared with UVB-irradiated cells.

## 3. Results

### 3.1. Identification of Prinsepiae Nux Extract as an NRF2 Activator

To identify NRF2 activators for skin protection, we performed high-throughput screening (Figure S1a) of the Shen Nong Ben Cao Jing Top-Grade Drugs Library, which contains 816 fractions prepared from 60 top-grade drugs with an HaCaT cell stably carrying an NRF2-driven luciferase reporter gene (HaCaT/ARE cells) [13]. HaCaT/ARE cells were treated with 816 fractions at 100 μg/mL for 18 h. Extracts that could increase NRF2 activity to 908% were considered hits (Figure S1a,b). A total of 11 hits were selected for validation and were further tested at a broader range of concentrations. Based on the level of maximum induction, the top three NRF2 activators were the extract fractions of Prinsepiae Nux, *Carpesium abrotanoides* L., and Eucommiae Cortex (Figure S1c). Prinsepiae Nux has been used to treat inflammation and redness of the eyes, relieve heat, and nourish blood and liver. Thus, it is interesting to investigate the protective effects of Prinsepiae Nux extract against skin aging.

**Figure 1:**
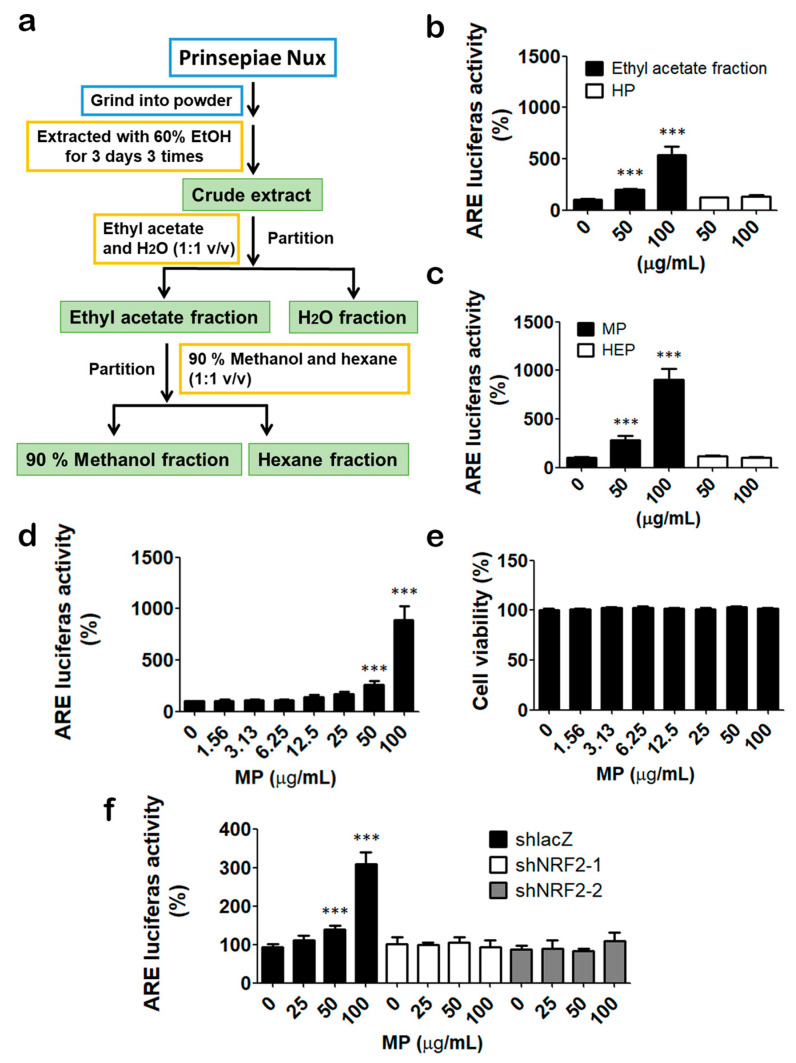
Prinsepiae Nux fraction increased NRF2 activity. (**a**) Flowchart of solvent partition. (**b**,**c**) HaCaT/ARE cells were treated with extracts from ethyl acetate fraction, H_2_O-partitioned (HP) fraction, 90% methanol (MP) fraction, and n-hexane (HEP) fraction for 18 h for reporter assay. (**d**) HaCaT/ARE cells were treated with indicated concentrations of MP fraction for 18 h for reporter assay. (**e**) HaCaT cells were treated with indicated concentrations of MP fraction for 72 h for cell viability assay. (**f**) Control (shLacZ) and NRF2 knockdown (shNRF2-1 and shNRF2-2) cells were treated with MP fraction for 18 h for reporter assay. DMSO solvent control was used as 100%. Data are presented as mean ± SD from three independent experiments. The asterisk (*) indicates a significant difference from the solvent control cells. (*** *p* < 0.001, one-way ANOVA).

To gain more insight into the NRF2-activating effect of Prinsepiae Nux, we prepared different solvent-partitioned fractions as described in the Section 2 (Figure 1a) and evaluated their NRF2-activating activity. The ethyl acetate fraction can induce NRF2 activity up to 500%. In comparison, the H_2_O-partitioned (HP) fraction has no significant effect (Figure 1b). Based on this result, ethyl acetate fraction was collected for further partition with 90% methanol (MP) and *n*-hexane (HEP). MP fraction can induce more than 900% NRF2 activity at a concentration of 100 μg/mL (Figure 1c). The results indicate that the NRF2-activating components were concentrated in the 90% methanolic partitioned fraction, which was then used for further examination. Moreover, MP fraction increased NRF2 activity in a dose-dependent manner (Figure 1d) with no cytotoxicity within 100 μg/mL (Figure 1e). In addition, depletion of NRF2 by shRNA in HaCaT/ARE reporter cells abolished the induction of luciferase activities by MP fraction (Figure 1f), which indicated that the effect of MP fraction is NRF2-dependent.

### 3.2. MP Fraction Activates NRF2 Signaling Pathway in HaCaT Cells

**Figure 2:**
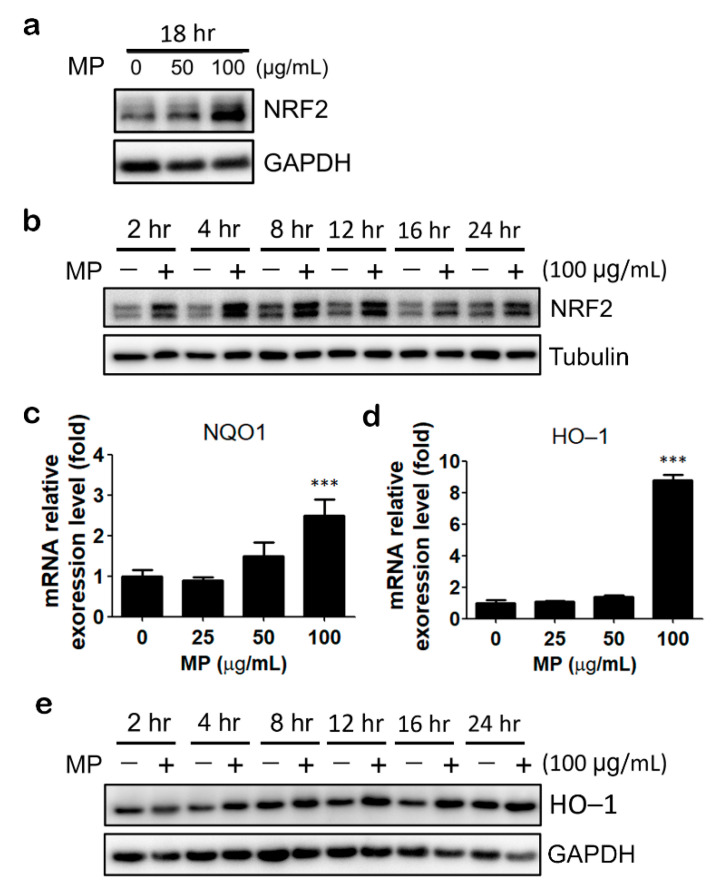
MP fraction activated NRF2 signaling pathway in HaCaT cells. (**a**) HaCaT cells were treated with MP fraction for 18 h. Cells were harvested for immunoblot analysis. (**b**) HaCaT cells were treated with MP fraction treatment for 2 to 24 h. The protein expression level of NRF2 was determined by immunoblot. (**c**,**d**) HaCaT cells were treated with MP fraction for 18 h for QPCR assay. DMSO solvent control was used as 1. Data are expressed as normalized mRNA levels and presented as mean ± SD. (**e**) HO-1 protein expression was determined by immunoblot following MP fraction treatment for 2 to 24 h. The asterisk (*) indicates a significant difference from the solvent control cells. (*** *p* < 0.001, one-way ANOVA).

Next, we evaluated the effect of MP fraction on NRF2 signaling. MP fraction increased NRF2 protein level in a concentration- and time-dependent manner in HaCaT cells (Figure 2a,b). The protein expression level of NRF2 increased in 2 h and reached the highest in 4 to 12 h (Figure 2b). Next, the expression levels of NRF2 downstream targets, namely NQO1 and HO-1, were determined. MP fraction substantially increased the mRNA expression of HO-1 and NQO1 in a dose-dependent manner (Figure 2c,d). Moreover, a prominent upregulation of HO-1 protein expression was noticed after 12 to 16 h of MP fraction treatment (Figure 2e).

### 3.3. Pre-Treatment of MP Fraction Could Induce Prolonged Activation of NRF2

**Figure 3:**
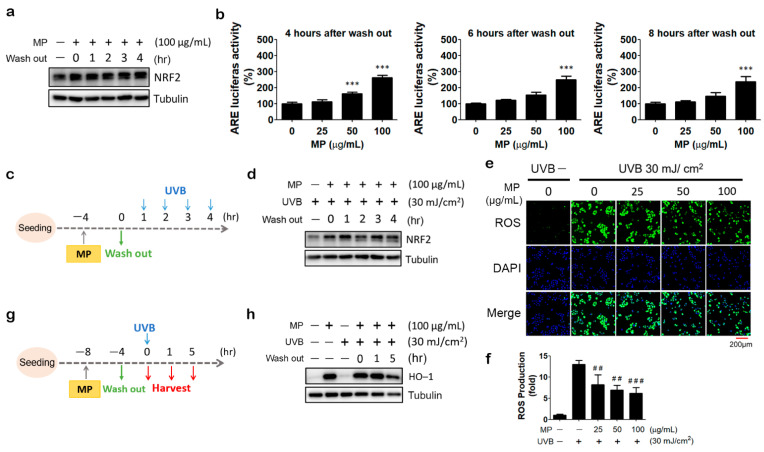
MP fraction induced prolonged activation of NRF2 and reversed UVB’s effects on NRF2, HO−1, and ROS level in HaCaT cells. (**a**) Cells were treated with MP fraction for 4 h and washed out for 0 to 4 h, then cells were harvested for immunoblot. (**b**) Cells were incubated with MP fraction for 4 h and washed out for 4, 6, and 8 h for reporter assay. (**c**,**d**) Cells were treated with MP fraction for 4 h and washed out for 0 to 4 h before UVB irradiation, then cells were harvested for immunoblot. (**e**,**f**) Cells were treated with MP fraction for 4 h and washed out for 4 h prior to UVB irradiation for intracellular ROS detection assay. (**g**,**h**) Cells were pre-treated with MP fraction for 4 h and washed out for 4 h before UVB irradiation. One to five hours after UVB exposure, cells were harvested for immunoblot. Data are presented as mean ± SD from three independent experiments. The statistical significance was defined as *** *p* < 0.001 compared with solvent control cells; ## *p* < 0.01, ### *p* < 0.001 compared with UVB-irradiated cells.

We intended to investigate how long MP fraction’s effects remain after being washed away. We treated HaCaT cells with MP fraction for 4 h because it reaches the maximum induction of NRF2 accumulation; then, the cells were washed and cultured in the medium without MP fraction for up to 4 h. The immunoblot result shows that the NRF2 protein retained the same level 4 h after MP fraction washout (Figure 3a). Furthermore, results from the reporter assay show a profound and persistent induction of NRF2 activity even at 8 h after MP fraction washout (Figure 3b).

### 3.4. MP Fraction Pre-Treatment Abolished NRF2 Depletion and Reduced ROS Production in UVB-Irradiated HaCaT Cells

As the most common extrinsic aging factor that causes skin aging, UVB is well-known to increase the production of ROS in keratinocytes [6]. Several reports showed that UVB exposure increases ROS levels in HaCaT cells [15,16]. It is also found that UVB irradiation induced NRF2 degradation via the activation of TRPV1 channels [17]. In agreement with these observations, we showed that exposure to 30 mJ/cm^2^ of UVB immediately and dramatically increased intracellular ROS generation in HaCaT cells. The ROS level was at the highest level for one hour. This was followed by a gradual decline over the next few hours (Figure S2a,b). Furthermore, we found that UVB irradiation caused a substantial decrease in NRF2 protein expression level, which could last for at least 8 h (Figure S2c). Since the NRF2 activating effect of MP fraction could maintain for a couple of hours after its removal, we next tested whether the pre-treatment of MP fraction could protect keratinocytes from UVB-induced oxidative stress in the washout period. HaCaT cells were treated with MP fraction for 4 h and exposed to UVB (30 mJ/cm^2^) after 0 to 4 h of MP fraction removal (Figure 3c). As shown in Figure 3d, pretreatment of MP fraction maintained the NRF2 protein level in UVB-irradiated HaCaT cells, even at 4 h after MP fraction washout. Moreover, under the same treatment condition, a significant and concentration-dependent decrease in UVB-induced ROS production was observed in MP fraction pretreated HaCaT cells (Figure 3e,f). To confirm the activation of NRF2 signaling after MP fraction removal and UVB irradiation, HaCaT cells were treated with MP fraction by 4 h incubation/4 h washout protocol and followed by UVB irradiation. The protein expression of HO-1 was determined at 0, 1, and 5 h after UVB exposure (Figure 3g). As shown in Figure 3h, the pre-treatment of MP fraction dramatically increased HO-1 expression, and the effect can last for at least 5 h after UVB exposure.

### 3.5. MP Fraction Ameliorated UVB-Induced Inflammatory Reaction

**Figure 4:**
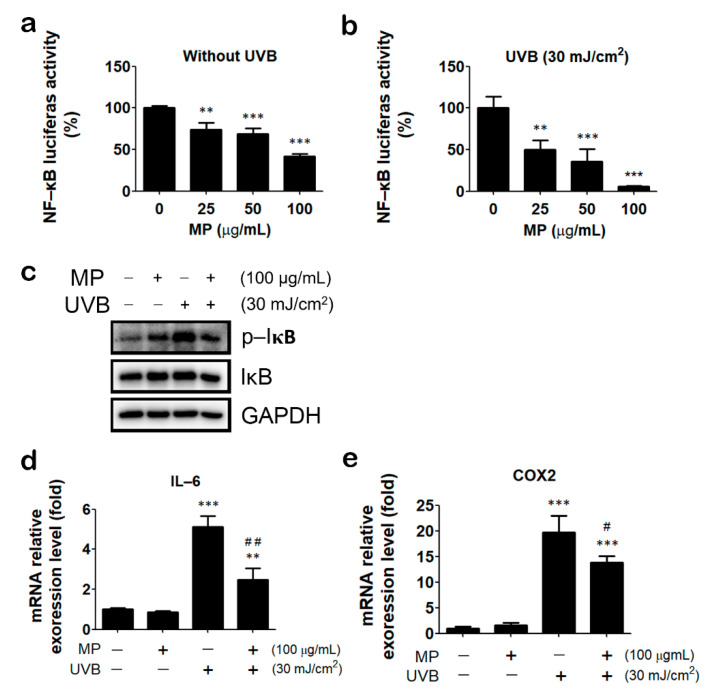
MP fraction alleviated UVB−induced activation of NF-κB signaling. (**a**) MP fraction was incubated with HaCaT/KBR cells for 4 h and washed out for 4 h with reporter assay. (**b**) HaCaT/KBR cells were treated with MP fraction as described in (**a**) and followed by UVB irradiation and reporter assay. (**c**) HaCaT cells were pre-treated with MP fraction for 4 h and then washed out for 4 h. Then, cells were exposed to UVB and harvested for immunoblot. (**d**,**e**) HaCaT cells were pre-treated with MP fraction for 4 h and then washed out for 4 h before UVB exposure, and incubated in serum-free DMEM for 18 h, then cells were harvested for QPCR. Data are presented as mean ± SD from three independent experiments. The statistical significance was defined as ** *p* < 0.01 and *** *p* < 0.001 compared with solvent control cells; # *p* < 0.05, ## *p* < 0.0 compared with UVB-irradiated cells.

The NF-κB signaling pathway is known to be activated by UV irradiation and involved in the process of skin aging [18]. Thus, we next tested the effects of MP fraction on UVB-induced NF-κB signaling. Our result shows that MP fraction can reduce NF-κB activity in HaCaT cells stably carrying an NF-κB driven luciferase reporter gene (HaCaT/KBR cells), even without UVB irradiation (Figure 4a). Moreover, HaCaT/KBR cells were pre-treated with MP fraction according to the aforementioned 4 h incubation/4 h washout protocol before UVB exposure. Cells were then harvested for determining the NF-κB activity, the phosphorylation of IκB, and the expression of NF-κB target genes, including IL-6 and COX-2. The results reveal that UVB increased the phosphorylation of IκB and mRNA expression of IL-6 and COX-2, which can all be reduced by MP fraction pre-treatment (Figure 4b–e). These findings indicated that MP fraction could protect keratinocytes from UVB-induced inflammatory reactions.

### 3.6. Characterizing the Constitutional Variation between Prinsepiae Nux Fractions Using Tandem Mass Spectroscopy and Molecular Networking Approach

**Figure 5:**
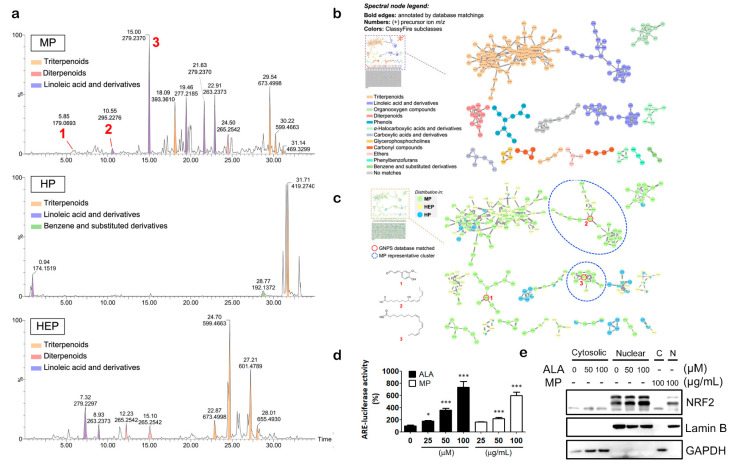
The chemical profiles and molecular evidence indicated that ALA could be an active component in Prinsepiae Nux. (**a**) The UPLC-MS total ion chromatogram was performed in positive mode. (**b**,**c**) The automated chemical classification was analyzed using ClassyFire from the GNPS platform. The annotations of constituents were carried out automatically through GNPS library matches. (**b**) The classical molecular network spectral nodes are colored according to ClassyFire classes; (**c**) the representative distributions of constituents in the Prinsepiae Nux extracts. (**d**) HaCaT/ARE cells were treated with indicated concentrations of ALA or MP fraction for 18 h for reporter assay. DMSO solvent control was used as 100%. Data are presented as mean ± SD from three independent experiments. (**e**) HaCaT cells were incubated with ALA or MP fraction for 4 h and harvested for immunoblot. Data are presented as mean ± SD from three independent experiments. The asterisk (*) indicates a significant difference from the solvent control cells. (* *p* < 0.05, *** *p* < 0.001, one-way ANOVA).

The main values of herbal medicine are complexes and a specific combination of ingredients, which are the fine inheritances of the ancients’ experience. Therefore, thoroughly clarifying the chemical and pharmacological properties among these complexes is currently the primary topic of herbal medicine translational research. Herein, we took advantage of mastering spectroscopic techniques, including “chromatographic bioassay-guided fractionation” and “molecular networking (MN)”, to analyze and refine the relationships between the chemical diversity and bioactivities of Prinsepiae Nux. In order to further address these constituent-dependent bioactivity differences between the aforementioned Prinsepiae Nux fractions (HP, MP, and HEP), an ultra-performance liquid chromatography quadrupole time-of-flight mass spectrometry (UPLC-QTOF-MS) was applied to perform the qualitative analysis. The acquired MS/MS fragmentation (MS^2^) data were then interpreted through ClassyFire chemical classification and annotated based on the GNPS MN database, resulting in the identification (Figure 5a) of several major chemical classes (linoleic acids, triterpenoids, diterpenoids, and benzenes), as well as three known metabolites: coniferaldehyde (1) [19], 9-hydroxy-10,12,15-octadecatrienoic acid (2) [20], and α-linolenic acid (3) [21].

In addition, the visualized MN explored the in-depth metabolomic diversity of constituents from MP fraction based on ClassyFire classification (Figure 5b). The majority of “linoleic acid” metabolites were revealed in MP fraction (the most active fraction) (Figure 5c). Since the parent ions labeled with linoleic acid (purple colored) were also found to be intense peaks in MP fraction but not HP fraction and HEP fraction (Figure 5b), it was suggested that the linoleic acid derivatives may act as indicative roles in the NRF2 activating activity of MP fraction. The molecular ion peak with a retention time of 15 min was identified as α-linolenic acid (ALA). Moreover, results from the reporter assay show that ALA could induce NRF2 activity significantly in a concentration-dependent manner (Figure 5d). In addition, the nuclear translocation of NRF2 was observed in ALA-treated HaCaT cells (Figure 5e). These findings suggested that ALA could contribute to the NRF2-activating effect of MP fraction.

## 4. Discussion

Numerous studies have confirmed that UVB induces excessive production of ROS in cells, which in turn causes irreversible damage to cells and leads to dermatitis, wrinkles, photoaging, and skin cancer. In this study, we identified an extract fraction from Prinsepiae Nux as an NRF2 activator by high-throughput screening with the Shen Nong Ben Cao Jing Top-Grade Drugs Library. A bioassay-guided fractionation experiment revealed that NRF2-activating components were concentrated in the 90% methanol (MP) fraction. MP fraction treatment led to NRF2 accumulation and increases in the mRNA expression of HO-1 and NQO1. It is worth noting that the NRF2-activating effect of MP fraction can maintain for 8 h after MP fraction removal. Pretreatment of MP fraction alleviated UVB-induced NRF2 depletion, ROS generation, and inflammatory reaction. Our findings suggested that the methanolic fraction extract of Prinsepiae Nux has the potential to protect keratinocytes from UVB-induced damage by activating NRF2 signaling.

Prinsepiae Nux or “ruiren” is a traditional Chinese medicine that has effects on dispelling wind and heat and nourishing the liver for improving eyesight. Both the dried kernel of *Prinsepia uniflora* Batal. and *Prinsepia uniflora var. serrata* Rehd. can be the original materials of Prinsepiae Nux [11]. Prinsepiae Nux is classified as a top-grade medicine in the “Shen Nong Ben Cao Jing”, the earliest classic Traditional Chinese Medicine pharmacology. Top-grade drugs are known to have superior health benefits and therapeutic potentials without toxicity, and are thus often used for health preservation [22]. In agreement with the notion that supplements for health preservation often have a high antioxidant activity [23], we demonstrated that the methanolic fraction extract of Prinsepiae Nux could activate NRF2, which is the master regulator of cellular antioxidant response. To the best of our knowledge, we are the first to study the effects of Prinsepiae Nux on NRF2 signaling in HaCaT cells and to show its protective function against UVB-induced ROS production and inflammatory reaction in HaCaT cells.

To date, only a few reports describe chemical constituents of the kernel of *P. uniflora* or Prinsepiae Nux. A research team in China first isolated 15 compounds from Prinsepiae Nux, including two flavonoids: kaempferol, and quercetin; three sterols: β-sitosterol, daucosterol, stigmast-4-ene-3β,6β-diol; five phenolic compounds: vanillic acid, protocatechuic acid, 1-(4-hydroxy-3-methoxy)-phenyl-1,2,3-propanetriol, ferulaldehyde (coniferaldehyde), gallic acid; two triterpenoids: ursolic acid, diploptene; one neolignan: balanophonin; succinic acid; and N-acetyl-glutamic acid [24,25]. Zhou and colleagues later identified two alkaloid galactosides: 5-[(α-D-galactopyranosyloxy) methyl]-1H-pyrrole-2-carbaldehyde and 6-[(α-D-galactopyranosyloxy) methyl]-3-pyridinol from water and n-BuOH extracts of Prinsepiae Nux, respectively [26]. Wu and colleagues collected kernels of *P. uniflora* from fresh fruits. The kernel was shelled and extracted by ethyl ether. They used gas chromatography-mass spectrometry to analyze the chemical profile of the kernel of *P. uniflora*. They identified 60 compounds in the extract. The majority of components are sesquiterpenes (61.24%), β-bourbonene, β-caryophyllene, τ-muurolol, α-copaene, palmitic acid, and margaric acid [27]. Among them, several compounds such as kaempferol [28], quercetin [29], β-sitosterol [30], ursolic acid [31], coniferaldehyde [32], vanillic acid [33], protocatechuic acid [34], and β-caryophyllene [35] have been reported to activate the NRF2 signaling pathway. Here, the bioactivity-guided partial purification assay showed that only the MP fraction but not HP fraction or HEP fraction can induce NRF2 activity markedly. MS/MS and MN analyses revealed that linolenic acid derivatives were enriched in MP fraction but not in other extracts without NRF2-activating activity. Surprisingly, we only identified coniferaldehyde from MP fraction. Notably, the most abundant ion peak in MP fraction was identified as ALA, which was further proved to activate NRF2 signaling in HaCaT cells. These findings agree with previous studies that ALA or ALA-containing extracts activate NRF2 and increase the expression of its target genes [36,37,38,39].

MS/MS shows the high efficiency of compounds characterization and quantitative analysis, in particular the multiple reaction monitoring (MRM) experiment that is designed to detect the specific MS/MS fragmentations from the precursor ions based on multi-quadrupole MS spectroscopy [40]. We hereby performed an MRM experiment to develop a rapid and sensitive method for the qualitative and quantitative quantification of ALA (**3**), the major linoleic acid derivative in MP fraction. By the developed quantitative protocols, the amount of ALA (**3**) was analyzed to be 0.68% (Method S1 and Figure S3). This amount is equivalent to ~2 μM of ALA when cells were treated with 100 μg/mL of MP fraction. However, we did not observe notable induction of NRF2 activity by ALA at a concentration below 5 μM (data not shown). Interestingly, we found that γ-linolenic acid (GLA), a positional isomer of ALA, could activate NRF2 and is more potent than ALA (Figure S4). This observation, together with the results of molecular networking analysis, suggests that other novel linoleic acid derivatives in MP fraction could act together to activate NRF2. Nevertheless, we do not rule out the possibility that triterpenoids, another major class of chemicals in MP fraction, are responsible for its NRF2 activation activity. Thus, conventional purification, isolation, and structure elucidation studies are undertaken to unveil the identities of active components in MP fraction.

In recent years, linoleic acid and linoleic-acid-containing oils have become increasingly popular in the cosmetic industry due to their beneficial properties on the skin. Several researchers point out that linoleic acid has anti-inflammatory, acne reductive, moisture retentive, and skin-lightening properties when applied topically on the skin [41,42,43,44]. In addition to topical application, Takemura and colleagues reported that an ALA-rich diet, but not the linoleic-acid-rich diet, suppressed UVB-induced skin injury and prostaglandin E2 (PGE2) production in hairless mice [45]. It was believed that omega-3 polyunsaturated fatty acids (PUFAs), such as ALA, could protect skin by maintaining skin-barrier function [46,47,48]. Nevertheless, Choi and colleagues reported that cold-pressed perilla (*Perilla frutescens* L.) oil, which has a remarkable quantity of linoleic acid, significantly reduced UV-induced ROS production, cellular damage, and cutaneous photoaging [49]. Moreover, Hwang and colleagues showed that linoleic acid is the main component of *Coriandrum sativum* L. (coriander leaf, cilantro; CS) leaf extract (CSE). They further demonstrated that CSE prevented skin photoaging by reversing UVB-induced alteration in procollagen type I production and MMP-1 expression in dermal fibroblasts and in hairless mice. Their results also imply the involvement of transcription factor activator protein-1 (AP-1) inhibition in the protective effects of CSE [50]. Here, we showed that MP fraction, enriched in linoleic acid derivatives, could also reduce UVB-induced ROS production and inflammatory reaction, and, for the first time, could activate NRF2 signaling in HaCaT keratinocyte cells. Moreover, we demonstrated that the effect of MP fraction can last for 4 to 8 h after it was washed away. Taken together, these findings indicated that ALA might protect skin via multiple mechanisms. Furthermore, our findings suggested that the methanolic fraction extract of Prinsepiae Nux (MP) has the potential for further development as an ingredient for skin care cosmetic products.

## 5. Conclusions

This study proved for the first time that the extract of Prinsepiae Nux can protect human keratinocytes (HaCaT cells) from UVB-induced oxidative damage, and the protective effect is closely associated with the activation of the NRF2 signaling pathway. Traditionally, Prinsepia Nux has been used to treat inflammation and redness of the eyes, such as acute conjunctivitis or acute keratitis, and can also relieve heat and nourish blood and liver. Corneal injury is also a common illness caused by UVB overexposure. In conclusion, our findings suggest the potential applications of Prinsepiae Nux for the development of pharmaceutics and cosmetics for the treatment or prevention of UVB-induced eye injury or skin aging.

## Data Availability

The data are contained within the article and Supplementary Materials.

## Supplementary Materials

The following supporting information can be downloaded at: https://www.mdpi.com/article/10.3390/antiox11091755/s1, Method S1: Qualitative and quantitative analysis using Ultra-performance Liquid Chromatography-Tandem Mass Spectrometry (UPLC-MS/MS); Table S1: The sequence of primers used for quantitative real-time PCR amplification; Figure S1: In Shen Nong Ben Cao Jing Top-Grade Drugs Library, the fraction of Prinsepiae Nux was selected as the most promising NRF2 activators; Figure S2: UVB induced the ROS production and the NRF2 depletion in HaCaT cells; Figure S3: The developed of qualitative and quantitative protocol of α-linolenic acid (3) from Prinsepiae Nux using multiple reaction monitoring (MRM) experiment; Figure S4: γ-linolenic acid (GLA) could activate NRF2 activity.
