# Supplementary material for: Prinsepiae Nux Extract Activates NRF2 Activity and Protects UVB-Induced Damage in Keratinocyte
Source: Antioxidants (Basel). 2022 Sep 5;11(9):1755. doi: 10.3390/antiox11091755 (PMC9495439; doi:10.3390/antiox11091755)
Supplement: Supplementary file 1 [file antioxidants-11-01755-s001.zip › antioxidants-1845195-supplementary.pdf]

Supplementary materials for

Prinsepiae Nux extract activates NRF2 activity and protects UVB-induced damage in keratinocyte

Shih-Han Wang, Yi-Siao Chen, Kuei-Hung Lai, Chung-Kuang Lu, Hsun-Shuo Chang, Ho-Cheng Wu, Feng-Lin Yen, and Chia-Hung Yen*

Content:

Supplementary Method S1.
Supplementary Table S1.
Supplementary Figure S1.
Supplementary Figure S2.
Supplementary Figure S3.
Supplementary Figure S4.

Supplementary Method S1. Qualitative and Quantitative analysis Using Ultra-performance Liquid Chromatography-Tandem Mass Spectrometry (UPLC-MS/MS)

A Shimazu Nexera X2 UPLC system (Shimazu, Kyoto, Japan) was applied for the qualitative and quantitative analysis of Prinsepiae Nux extract. Liquid chromatography was carried out utilizing a Thermo Hypersil GOLD C18 (1.9 µm, 2.1 mm × 100 mm) column (Waltham, MA, USA). The mobile phase was prepared by mixing acetonitrile (A, containing 0.1% formic acid) and water (W, containing 0.1% formic acid), the gradient sequence was executed as follows: 0.01–10 min, 50–55% A; 10.01–13 min, 50% A. The flow rate was fixed at 0.5 mL/min, the column temperature was maintained at 40 °C. To prepare the sample, 2 mg PU extract was dissolved in 1 mL of methanol and was filtered through a 0.45 µm membrane filter before loading into the UPLC column. The sample injection was implemented automatically with 5 µL volume per injection.

The production scan experiments (in the positive or negative mode) and multiple reaction monitoring (MRM) were performed using Shimazu LCMS-8045 mass spectrometry. The dwell time was set at 100 msec and the collision energy (CE) was set as -35V. The characteristic product ions of compound were picked for MRM settings and the calibration curves were then constructed by plotting the peak areas of ion currents versus five concentrations (10, 50, 100, and 250 ppm) of the standard compounds. All the acquired MS data were processed by LCMS LabSolutions software (Version 5.93, Shimazu, Kyoto, Japan).

Supplementary Table S1. The sequence of primers used for quantitative real-time PCR amplification.

Gene name	Gene bank ID	Primer Sequences (5'-3')
NQO1	NM_000903	F: TGCAGCGGCTTTGAAGAAGAAAGG
		R: TCGGCAGGATACTGAAAGTTCGCA
HO-1	NM_002133	F: GCCAGCAACAAAGTGCAAG
		R: GAGTGTAAGGACCCATCGGA
IL-6	NM_000600	F: ACTCACCTCTTCAGAACGAATTG
		R: CCATCTTTGGAAGGTTCAGGTTG
COX-2	NM_000963	F: GTTCCACCCGCAGTACAGAA
		R: AGGGCTTCAGCATAAAGCGT
GAPDH	NM_002046	F: GCAAATTCCATGGCACCGTCA
		R: TCCTGGAAGATGGTGATGGGA

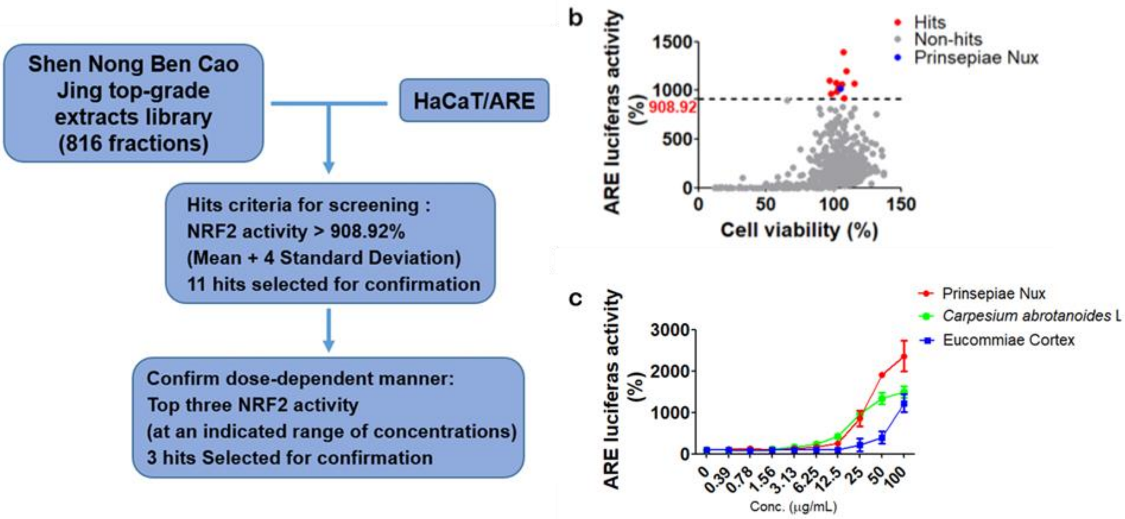

Supplementary Figure S1. In Shen Nong Ben Cao Jing Top-Grade Drugs Library, the fraction of Prinsepiae Nux was selected as the most promising NRF2 activators. (A) The flowchart of high-throughput screening (B) HaCaT/ARE cells were seeded in 96-well plates at 10,000 cells per well and treated with analytes at 100 μg/mL for 18 hours. The Y axis represents ARE luciferase activity in HaCaT/ARE cells, the X axis represents cell viability. Each dot in the figure represents a fraction, and red dots regard as hits. (C) HaCaT/ARE cells were treated with 3 selected fractions at indicated concentrations for 18 hours. ARE luciferase activity was calculated by normalizing luciferase activity with cell viability. DMSO solvent control was used as 100 % activity. Data are presented as mean ± SD, n = 3.

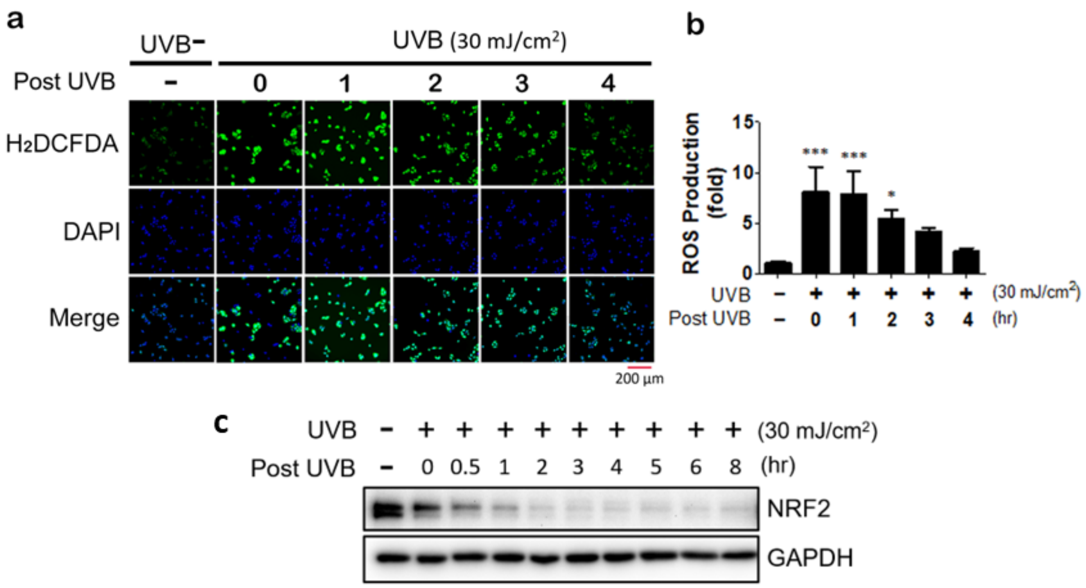

Supplementary Figure S2. UVB induced the ROS production and the NRF2 depletion in HaCaT cells. (A) HaCaT cells were exposed to 30 mJ/cm^2 of UVB and incubated in serium-free DMEM and placed in a 37 ºC incubator with 5% CO_2 for 0 to 4 hours. Then cells were harvested for intracellular ROS detection. (B) Quantified fluorescence intensity represented ROS production. (C) HaCaT cells were exposed to UVB (30 mJ/cm^2) and then incubated in serium-free DMEM and placed in a 37 ºC incubator with 5% CO_2 for 0 to 8 hours for immunoblot analysis. The asterisk (*) indicates a significant difference from the solvent control cells. (*$p<0.05$ and ***$p<0.001$, one-way ANOVA)

a

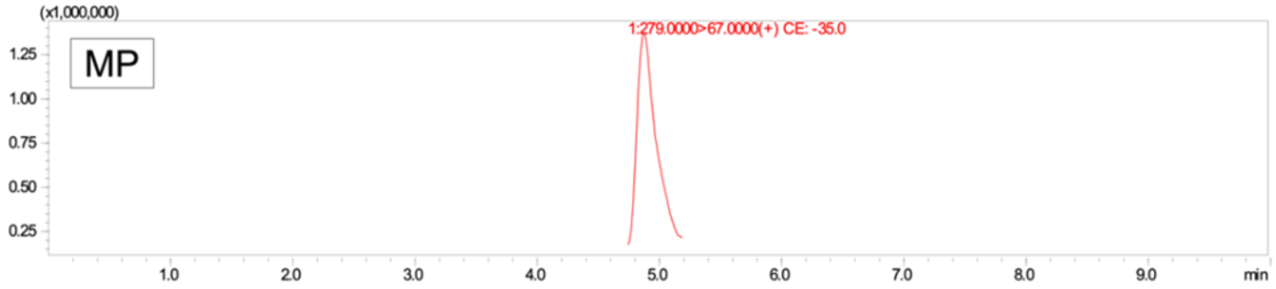

b

Compounds	Ion adduct	Precursor/ product[a] ion (*m/z*)	CE	Calibration curve	R^2	Amount (%)
α-linolenic acid	$[M + H]^+$	279/67	-35	Y = 168203X + 1E+06	0.9984	0.68

[a]The product ions were selected for MRM quantitative analysis.

Supplementary Figure S3. The developed of qualitative and quantitative protocol of α-linolenic acid (3) from Prinsepiae Nux using multiple reaction monitoring (MRM) experiment. (A) The product ions chromatogram; (B) The LC-MS/MS analysis method.

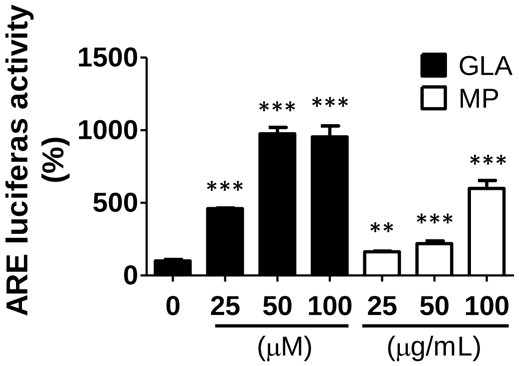

Supplementary Figure S4. γ-linolenic acid (GLA) could activate NRF2 activity. HaCaT/ARE cells were treated with indicated concentrations of GLA/MP for 18 hours for reporter assay. DMSO solvent control was used as 100 % activity. Data are presented as mean ± SD from three independent experiments. The asterisk (*) indicates a significant difference from the solvent control cells. (**p<0.01 and ***p<0.001, one-way ANOVA)
